# Degradation of anti-wear additives and tribological properties of engine oils at extended oil change intervals in city buses

**DOI:** 10.1038/s41598-025-12480-y

**Published:** 2025-07-26

**Authors:** Wojciech Gołębiowski, Rafał Krakowski, Grzegorz Zając

**Affiliations:** 1 Department of Power Engineering and Transportation, Faculty of Production Engineering, University of Life Sciences in Lublin, Głęboka 28, 20-612 Lublin, Poland; 2 Department of Ship Powerplants, Faculty of Marine Engineering, Gdynia Maritime University, Morska 81-87, 81-225 Gdynia, Poland

**Keywords:** Four-ball test, FTIR, XRF, City buses, Antiwear additives, Oil degradation, Mechanical engineering, Fluidics

## Abstract

The article presents the results of combined tribological and physicochemical studies of engine oils used in Autosan Sancity M12LF city buses at various mileages exceeding the manufacturer’s recommended oil change interval (60,000 km). Semi-synthetic oil of ACEA E4, E7, SAE 10W-40 class was analyzed, with samples taken from five buses with different degrees of interval exceedance (− 19% to + 35%). To address outlier influence, both classical Pearson and a more robust Spearman’s rank correlations were applied. While Pearson correlation suggested a very strong relationship (r = − 0.9946) between sulfur content and wear scar diameter, Spearman’s robust method confirmed a strong but more reliable association (r = − 0.810), indicating that classical correlation was inflated by outliers. A potential relationship was demonstrated between the degree of oil change interval exceedance and degradation of phosphate anti-wear additives. Anti-wear additive degradation follows a non-linear pattern and accelerates with successive exceedances of the recommended change interval. A degradation thresholds were identified: oil degradation becomes more pronounced after exceeding the interval by 10,000–15,000 km, while exceeding by more than 15,000–20,000 km results in potentialy increased engine component wear and elevated failure risk. The content of abrasive metals (Fe, Cu) in used oils significantly increased compared to fresh oil, with particularly high iron concentration (131.67 ppm) observed in the sample with the greatest interval exceedance. After exceeding the interval by 20,000 km, phosphate anti-wear additive depletion was 73% higher than when exceeding by 5000 km. The research results provide important information for bus fleet operators, allowing more precise determination of engine oil change intervals, which translates into extended engine life and reduced operating costs.

## Introduction

Engine oils play a key role in ensuring the proper functioning of internal combustion engines, serving as an essential component of the lubrication system. In addition to their primary lubricating function, engine oils are responsible for dissipating heat, protecting metal surfaces from corrosion, neutralizing acids formed during the combustion process, and maintaining the cleanliness of internal engine components. The effectiveness of engine oil directly affects energy efficiency, pollutant emissions, and engine durability, which is particularly important in the case of intensively used vehicles such as city buses.

However, engine oils inevitably undergo degradation during operation. This process occurs as a result of exposure to high temperatures, mechanical loads, contaminants, and combustion by-products^1^. This leads, among other things, to the deterioration of their anti-wear and anti-seizure properties^2,3^. In particular, performance additives such as ZDDP (zinc dialkyldithiophosphates), which are responsible for anti-wear protection, tend to become depleted over time, which can lead to accelerated wear of engine components^4^. Monitoring the condition of engine oil and understanding the mechanisms of its degradation are, therefore, key aspects in optimizing oil change intervals and minimizing operating costs^5^.

Contemporary research in the field of tribology focuses on seeking new solutions to improve the properties of engine oils^6–8^. One of the main directions is the development of advanced performance additives that can effectively counteract wear under boundary conditions^9^.

Agocs et al.^10^ demonstrated that the degradation of engine oil under operating conditions is complex in nature and leads to significant changes in its tribological properties. Studies by Padgurskas et al.^11^ confirmed that the boundary condition of engine oils can be determined by analyzing changes in tribological parameters during operation.

Despite significant advances in the field of performance additives, their effectiveness under real operating conditions remains a subject of discussion. Dörr et al.^12^ conducted a comprehensive chemical analysis of engine oil degradation in a passenger car, demonstrating the complexity of the processes occurring during operation. Masuko et al.^13^ studied changes in friction and wear related to ZDDP degradation under simulated engine oil degradation conditions.

An important aspect of research on used oils is also the analysis of abrasive metal content. Zając et al.^14^ used the XRF method to determine selected heavy metals in used engine oils. Stout et al.^15^ and Szyszlak-Bargłowicz i in.^16^ studied the concentration of metals in used engine oils depending on engine type and oil change interval.

Despite numerous laboratory studies, there is still a need to analyze real operating conditions, especially in vehicles operating in intensive cycles, such as city buses. Kral Jr et al.^17^ demonstrated that degradation and chemical changes in long-life oils occur in a non-linear manner during intensive operation in automotive engines.

As shown in the literature review, most authors rely on the use of only one of the engine oil research techniques, such as tribological studies, FTIR, or elemental analysis, which reveal specific oil parameters and element concentrations used to draw conclusions about the oil’s condition. Therefore, in this study, it was decided to combine several research methods (tribology, FTIR, XRF), which may represent a new approach to analyzing oil degradation. A better understanding of these mechanisms may enable more accurate decisions regarding the determination of oil change intervals, leading to an extended engine lifespan.

Hence the aim of the study was to analyze changes in the anti-wear properties of engine oil under typical operating conditions of city buses in the context of engine durability. In particular, the effects of oil degradation due to use were examined, as well as the extent to which the depletion of anti-wear additives affects the protection of engine components against wear.

The results may provide practical guidelines for fleet management, but further research is needed for confirmation. Our research expands existing knowledge by introducing a wide range of oil change interval exceedances (from − 19% to + 35% of the recommended mileage). Previous studies have also not shown such strong correlations between chemical composition and tribological properties.

## Materials and methods

### Materials

Six samples of semi-synthetic engine oil, including a sample of fresh oil, were used as the research material. The test samples consisted of one type of oil designated with the code name OP, in the SAE 10W40 viscosity grade and ACEA E4, E7 quality classes. The characteristics of the fresh oil sample, based on the authors’ tests, are presented in Table 1.

**Table 1:** Engine oil physicochemical properties.

	Properties	Unit	Values
Physicochemical properties	Kinematic viscosity at 40 °C	[mm^2^/s]	86
	Kinematic viscosity at 100 °C		14.7
	Viscosity index	–	169.5
	Total base number (TBN)	[mg KOH/g]	10.6
Elemental composition	Ca	[mg/kg]	4305
	P		960
	S		3806
	Zn		1376

Samples of oil used under real operating conditions were taken from five 12-m Autosan Sancity M12LF city buses. A detailed description of the vehicles from which the engine oil samples were collected is presented in Table 2.

**Table 2:** Engine parameters in the tested buses.

	Parameters
Type of bus	City
Type of fuel	Diesel
Year of production	2011–2013
Engine manufacturer	Iveco
Engine model	Cursor 8 F2BE3682B (Euro 4)
Displacement [cm^3^]	7790
Engine power / rpm	245 kW / 2400 rpm
Maximum torque/rpm torque	1400 Nm / 1100 rpm
Transmision / degrees	Automatic / 4
Recommended engine oil interval [km]	60,000

The average daily distance covered by the buses was 122 km. The buses were operated under typical urban conditions. Samples were collected during oil changes at various operational mileages. The oil change interval recommended by the vehicle manufacturer was 60,000 km; however, in one case it was shortened to 48,864 km (by about 19%), while in the other four buses it was extended by approximately 5, 10, 15, and 20 thousand kilometers, ranging from 10 to 35% above the recommended limit. The applied oil change intervals are presented in Table 3.

**Table 3:** Bus service maintenance data from municipal transport company.

		Bus ID				
		2378	2384	2400	2414	2420
Bus mileage	Overall [km]	874,729	868,964	130,719 (766,480)	704,454	77,240 (750,328)
	oil interval [km]	70,600	48,864	80,984	66,238	77,240
oil interval	start [date]	28.07.2022	07.05.2023	13.03.2023	02.08.2022	11.11.2023
	end [date]	22.09.2023	19.06.2024	06.06.2024	02.08.2023	28.06.2024
Mean value of operational availability during 1 year (from 05.2023–05.2024) [%]		80.8	77.4	83.5	72.8	86.2

Data from overall mileage showed in (..) presents mileage when entire engine or the engine head of the bus was replaced.

The average daily distance covered by the buses was 122 km. The buses were operated under typical urban conditions. Samples were collected during oil changes at various operational mileages. The oil change interval recommended by the vehicle manufacturer was 60,000 km; however, in one case it was shortened to 48,864 km (by about 19%), while in the other four buses it was extended by approximately 5, 10, 15, and 20 thousand kilometers, ranging from 10 to 35% above the recommended limit. The applied oil change intervals are presented in Table 3.

### Methods

The T-02U tribometer equipped with a four-ball head was used in the study. This device was used to assess the anti-seizure and anti-wear properties of oils and lubricants by national (PN-76/C-04147) and international standards (ASTM D 2783, ASTM D 2596, ASTM D 4172, ASTM D 2266, IP 239, DIN 51350, Fiat 50500, IP 300). The device enabled the simulation of friction and wear conditions characteristic of engines and machinery by applying a load to four balls immersed in the tested oil. During the experiment, the friction torque was recorded, which allowed for the evaluation of the lubricating properties of the tested samples.

A dynamic test was conducted using the apparatus to determine the load at which the seizure process begins, as well as the maximum pressure that can be applied before seizure occurs. The test involved gradually increasing the load on a set of four steel balls immersed in the tested oil until a sudden increase in resistance to motion was observed, indicating the initiation of the seizure process. The detailed test conditions are presented in Table 4.

**Table 4:** Test conditions.

Parameters	Condtions
Spindle rotational speed	500 ± 20 rpm
Load escalation rate	408.8 N/s (41.667 kgf/s) [4905 N] (500 kgf) per 100 revolutions of the upper ball,
Initial load on the friction node	0 N
Maximum load on the friction node	7200 N
Test time	18 s
Ambient temperature	20 ± 5 °C

The durability of the lubricating film, as well as the determination of the conditions for its breakdown and the onset of seizure, are assessed using:

- Seizure point (*P*_*t*_)—the value of the load at which, under specified test conditions, a sudden increase in friction resistance is observed, indicating a loss of lubricating ability;
- Limit seizure pressure (*p*_*oz*_)—the pressure in the friction pair under the applied load, calculated based on the average diameter of the scars formed on the lower balls during machine operation.

According to the applicable standard, each sample was analyzed ten times, and the final result was determined as the arithmetic mean of at least three measurements, with the difference between them not exceeding 10% of the mean value.

**Fig. 1:**
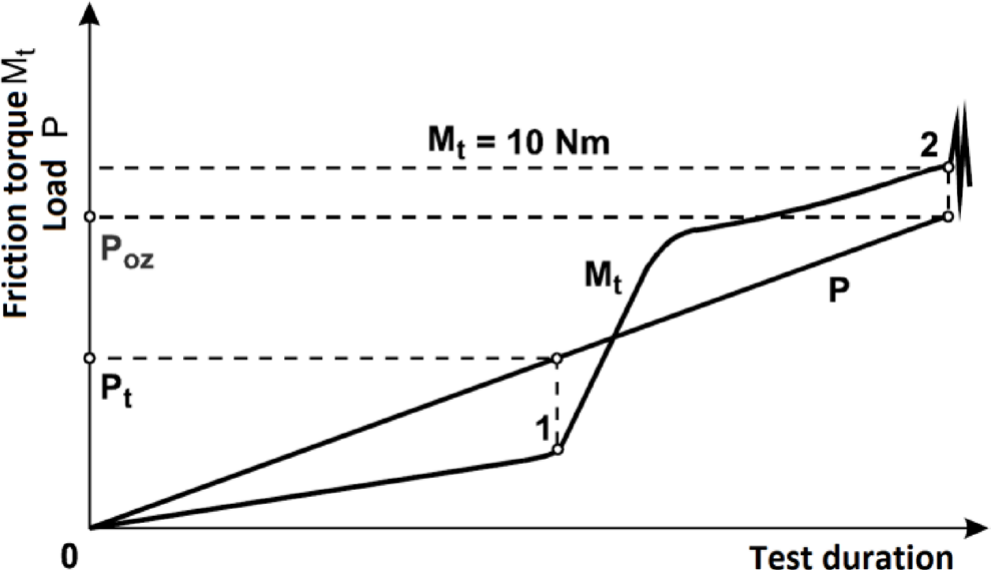
An example friction torque curve Mt obtained under conditions of continuous load increase P for a four-ball apparatus; 1—initiation of seizure, 2—seizure.

Figure 1 shows a model curve of friction torque Mt. The load P_t_ at point 1, where the friction torque begins to rise sharply, is called the seizure point.

The test method involves continuing to increase the load and recording the friction torque values until point 2 is reached, where seizure of the contact occurs, i.e., the critical friction torque value is exceeded. The load at this point is called the critical seizure load and is denoted by the symbol P_oz_. If no seizure is observed during the test run, the maximum achieved load on the contact is taken as the critical seizure load.

Seizure resistance was determined based on the seizure load index (P_t_) and the limit seizure pressure (*p*_*oz*_), which corresponds to the nominal pressure on the wear scar surface at the point of seizure of the contact or the end of the test (if seizure does not occur). It is calculated using the following formula:

1 $${p}_{oz}=0.52\frac{{P}_{oz}}{{d}^{2}}$$

where: P_oz_—limit seizure load [N]; d—average diameter of the wear scar on the balls [mm]; 0.52—coefficient resulting from the distribution of forces in the four-ball friction node.

The limit seizure pressure is an indirect indicator of the wear resistance of the tested contact operating under sliding friction conditions. A higher poz value indicates better effectiveness of the lubricant in protecting tribological surfaces after the lubrication limit has been exceeded.

**Fig. 2:**
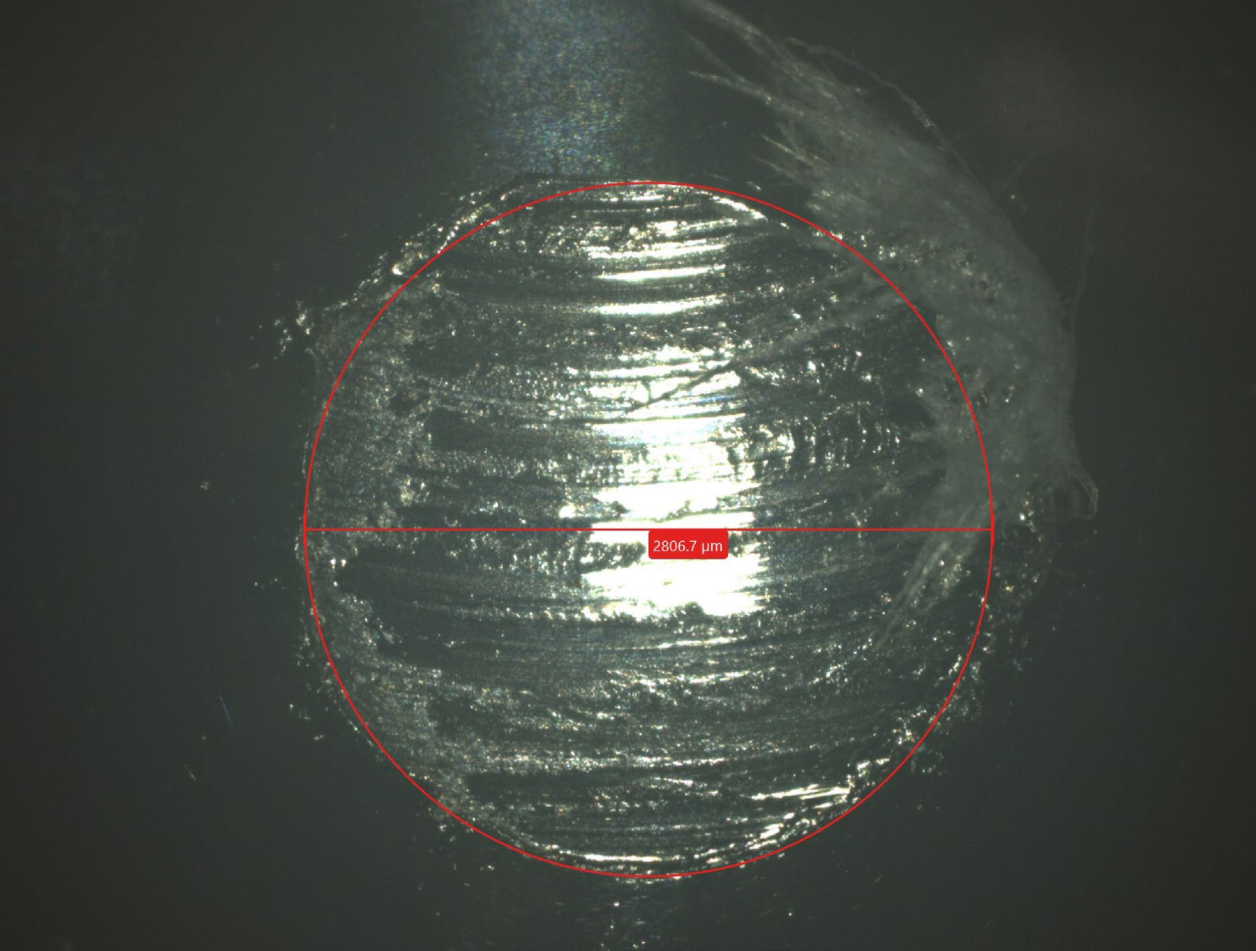
The wear scar diameter on the tested ball in Zaiss SmartZoom 5 microscopic magnification view.

The wear scar diameter on the test balls after tribological tests was measured using a Zeiss SmartZoom 5 digital measuring microscope. Example image of the ball after testing was shown in Fig. 2.

The analysis of the engine oil was carried out using an ERASPEC OIL FTIR mid-infrared spectrometer (Eralytics GmbH, Austria) to assess key parameters, including the degradation of ZDDP anti-wear additives, by ASTM E2412-10 and D7412 standards. The content of contaminants such as soot, as well as the decrease in phosphate anti-wear additive content, was evaluated. Soot content was determined as the degree of absorbance at two locations responsible for this type of contamination, namely at the absorbance bands at 2000 cm^−1^ and 3980 cm^−1^. The soot content in absorbance units was calculated as the average of the readings at 2000 cm^−1^ and 3980 cm^−1^, divided by 2. The content of phosphate anti-wear additives was determined by the recorded spectra of used oil were processed by subtracting the spectrum of fresh oil, enabling the creation of a differential spectrum. Negative signals result from the depletion of these additives, with new oil serving as the reference point. By measuring the minimum absorbance in the spectral range of 920–1030 cm^−1^, with the baseline defined by points P1 (920 cm^−1^) and P2 (1030 cm^−1^).

The second way of calculating the anti-wear additives, was the percentage of remaining anti-wear additives. This parameter was calculated based on the peaks and baselines for the used and fresh oil. The peaks were calculated as the area in the spectral range, defined by the limits P1 (990 cm^−1^) and P2 (1025 cm^−1^). Furthermore, the baselines, were assessed as sections passing through the two points whose values was the lowest, in the range defined by points P1 (905 cm^−1^) and P2 (1105 cm^−1^). The value was calculated based on the following formula:

2 $$P=100-\left(1-\frac{{P}_{used}}{{P}_{fresh}}\right)$$

The FTIR spectra of used oils were compared with the spectra of fresh oils to identify changes. These changes were determined using the intensity of the bands, measured by both their height and area.

Trace element analysis was performed using a Maxine HD multi-element analyzer (XOS, USA), which utilizes high-definition X-ray fluorescence (HDXRF) technology to accurately detect and quantify elemental concentrations. The primary focus was on measuring the levels of wear-related metals, specifically iron (Fe) and copper (Cu), which serve as indicators of engine component wear. In addition to these wear metals, the analysis also included elements commonly associated with engine oil additives. The concentrations of sulfur (S), zinc (Zn), and phosphorus (P)-key elements found in zinc dialkyldithiophosphate (ZDDP) anti-wear additives- were determined. Molybdenum (Mo), used as a friction modifier in many engine oils, and calcium (Ca), which acts as a detergent additive, were also measured to provide a comprehensive overview of the oil’s additive package and its degradation. Before conducting the measurements, the analyzer was carefully calibrated to ensure accuracy. For sample preparation, each oil sample was heated to 40 °C, and 1 ml was dispensed into a measurement cup lined with prolene film, which is suitable for XRF analysis. Each sample was analyzed three times, and the final results were calculated as the average of these measurements to ensure reliability and precision.

### Statistical analysis

To compare and determine the statistical significance of the results obtained from the analysis of individual oil parameters from five different buses, an ANOVA (Analysis of Variance) was conducted. The use of ANOVA allowed for the assessment of whether the observed differences in the measured parameters of the engine oils were due to actual differences between the vehicles or fell within the range of natural random variability. A statistical significance level of α = 0.05 was adopted.

Before applying ANOVA, the methodological assumptions regarding normality of distribution and homogeneity of variance were checked and verified. The Shapiro–Wilk test was used to determine whether each of the compared variable groups exhibited a normal distribution. The analysis showed that, for each group compared, there was no basis to reject the null hypothesis that the variables were normally distributed.

Next, Levene’s test was used to check for homogeneity of variance. The test yielded a p-value of less than 0.05, indicating that there was no basis to reject the null hypothesis of homogeneity of variance.

Next, a correlation matrix was constructed to provide insight into the interrelationships between various parameters of the used engine oils from all the tested city buses. The correlation analysis provided valuable information about the dependencies between engine oil degradation, the depletion of anti-wear additives, and the protection of engine components against wear.

Then, a regression analysis was conducted to determine the direct correlation between each oil property and the wear scar diameter.

Finally, due to the presence of outliers, robust methods such as Spearman’s rank correlations were also applied.

The statistical power of the presented analysis is limited, limitations is related to the small sample size which require further analysis. The authors are aware that the limited sample size presents a significant challenge in terms of generalizing the obtained results to a larger population. Our study was of a pilot nature and aimed to identify potential correlations between oil condition and the technical condition of the vehicle under real operating conditions. These results serve as a starting point for further, more extensive research, which will allow for a more comprehensive confirmation and generalization of the observed relationships.

## Results and discussion

### Tribological analysis—four-ball T-02U apparatus

The results of the seizure resistance of the tested oils, as well as the average values of the wear scar diameter on the balls, are presented in Table 5.

**Table 5:** Results of the tests carried out on the four-ball apparatus.

Operating condition	Sample code	Overall mileage [km]	Mileage on oil [km]	Seizure point *P*_*t*_ [N]	Limiting pressure of seizure * p*_*oz*_ [N/mm^2^]	Mean values of the wear scar diameter [mm]
				x̅ ± SD	x̅ ± SD	x̅ ± SD
New oil	OP	–	–	2800 ± 100	496.26 ± 43.66	2.747 ± 0.121
City	OP_2384	868,964	48,864	950 ± 132	40.32 ± 4.32	4.041 ± 0.053
	OP_2414	704,454	66,238	1833 ± 58	956.30 ± 12.01	1.980 ± 0.012
	OP_2378	874,729	70,600	1700 ± 0	1002.94 ± 65.53	1.932 ± 0.066
	OP_2420	77,240	77,240	1933 ± 58	960.38 ± 55.50	1.974 ± 0.057
	OP_2400	130,719	80,984	1233 ± 58	843.63 ± 31.33	2.107 ± 0.039

x̅ arithmetic average, *SD* Standard deviation.

The analysis of the oils’ seizure resistance revealed significant changes in their properties as a result of use. The fresh oil exhibited the highest seizure resistance, reaching a seizure point value of 2800 N, with an average wear scar diameter of 2.747 mm. Among the used oil samples, the greatest decrease in limiting pressure of seizure was observed in sample OP_2384, where the seizure point value was 950 N and the wear scar diameter was 4.041 mm (+ 47% compared to fresh oil). The best anti-wear properties were retained by samples OP_2378, OP_2420, and OP_2414, with seizure point values of 1700 N, 1933 N, and 1833 N, respectively, and relatively low wear scar diameters (1.932–1.980 mm). In most cases, a decrease in limiting pressure of seizure was recorded, with the values of limit load and ball wear diameters indicating varying degrees of oil degradation depending on mileage and the technical condition of the engine.

Empirical studies have shown that used engine oils can sometimes display lower wear rates and produce smaller wear scar diameters than fresh oils. This phenomenon is attributed to changes in the oil’s tribological properties during use, which may enhance its ability to withstand higher loads and offer improved protection against wear^18,19^.

The recommended oil change interval was 60,000 km; however, in one case, the oil was changed earlier (− 19%), while in the remaining vehicles, the interval was extended (from + 10% to + 35%). The results showed that extending the oil’s service life did not always lead to an immediate loss of its properties. Some samples (such as OP_2420) retained relatively good parameters despite a longer service period. In contrast, in the case of sample OP_2384, where the recommended oil change interval was not exceeded, significant degradation of lubricating properties occurred.

**Fig. 3:**
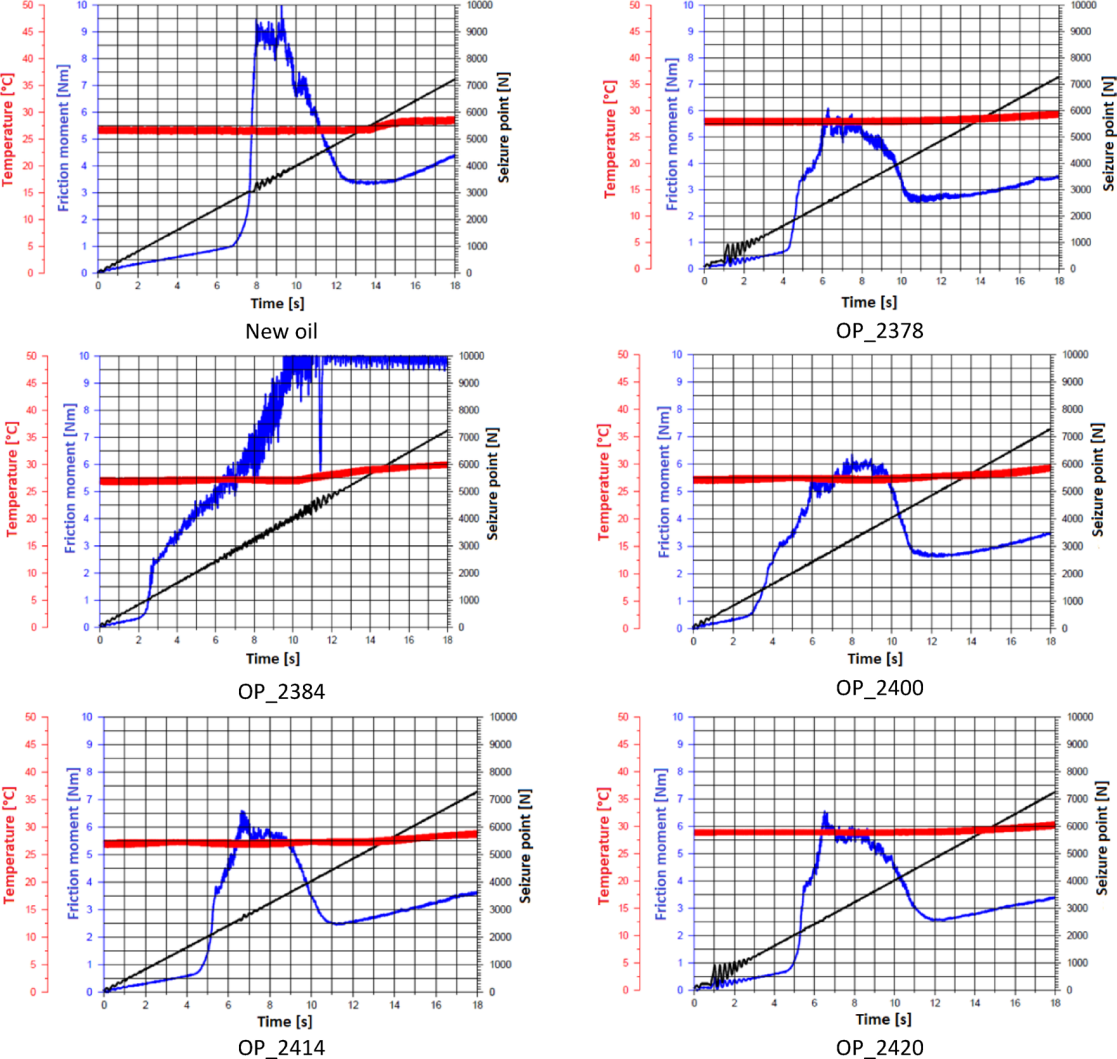
The course of friction torque and temperature with increasing load on the friction node of the tested engine oils.

Figure 3 presents examples of changes in friction torque and temperature as a function of increasing the load on the friction pair for the tested engine oils.

The friction torque curves reveal key stages of the wear process- from initial boundary lubrication, through gradual weakening of the oil film, to the rupture of the boundary layer and a sudden spike in friction torque leading to ball seizure. The final phase represents oil film renewal, characterized by friction stabilization with an upward trend. Sample OP_2384 is an example of significant tribological degradation, where high load without a clear limit seizure point P_oz_ may result from a complete loss of lubricating properties. A stable, high friction torque indicates dry friction without effective lubrication, while the largest wear scar diameter confirms a degradation of anti-wear additives. For the fresh oil, the lubrication process was the most stable: the boundary layer ruptured after a longer period, and friction torque increased gradually, reaching its maximum value under higher load. In contrast, for used oils, the boundary layer failed earlier, resulting in a faster rise in friction torque and greater wear severity. Specifically, sample OP_2378: The boundary layer ruptured faster than in fresh oil. Sample OP_2384: Exhibited the fastest boundary layer failure, with friction torque reaching the highest value among all tested samples, indicating significant degradation of lubricating properties. Samples OP_2400, OP_2414, and OP_2420: Showed varying levels of lubricating film stability. In some cases, partial regeneration of the boundary layer delayed seizure, but in most instances, friction torque surged abruptly, signaling advanced wear processes. The results highlight how oil degradation impacts boundary layer integrity and wear progression under load.

Tribological analysis revealed a clear trend of degradation in the lubricating properties of the oil with increasing operational use, where fresh oil achieved the highest seizure point (2800 N) with a characteristic increase in friction torque to a maximum (~ 9.5 Nm), followed by a sharp drop signaling seizure. Used oils exhibited a systematic reduction in seizure points P_t_ to values of 950–1933 N, with a typical mechanism: an increase in friction torque, reaching a maximum, followed by a sharp drop and stabilization at a lower level. The OP_2384 case was an extreme example of degradation, where, despite the absence of a classic limit seizure point (no sharp drop in friction torque), the sample exhibited continuous high friction (~ 9–10 Nm) at a load exceeding 7300 N and the largest seizure diameter of the balls (4.041 mm), unequivocally indicating a complete loss of effective lubrication. The overall trend confirms that progressive oil degradation leads to a systematic reduction in seizure resistance, with possibly significant engine wear (OP_2384).

### Elemental analysis—HD Maxine XRF

The analysis of abrasive metal concentrations in the oils, presented in Table 6, allowed for the assessment of engine wear intensity.

**Table 6:** Concentration of abrasive metals in the tested oils.

Operating condition	Sample code	Overall mileage [km]	Mileage on oil [km]	Cu [ppm]	Fe [ppm]
				x̅ ± SD	x̅ ± SD
New oil	OP	–	–	0	1.27 ± 0.04
City	OP_2384	868,964	48,864	4.48 ± 0.09	76.14 ± 0.06
	OP_2414	704,454	66,238	2.14 ± 0.14	75.87 ± 0.58
	OP_2378	874,729	70,600	11.30 ± 0.10	62.75 ± 0.83
	OP_2420	77,240	77,240	5.26 ± 0.17	131.67 ± 0.10
	OP_2400	130,719	80,984	9.30 ± 0.02	96.37 ± 0.81

x̅- arithmetic average, *SD* Standard deviation.

Iron (Fe) concentration in fresh oil was 1.27 ppm, while in used oil samples, it increased to a range of 62.75–131.67 ppm, indicating progressive degradation of steel engine components. The highest Fe concentration was observed in sample OP_2420 (131.67 ppm), suggesting intense engine wear in this vehicle. The difference in iron content between exceeding the oil change interval by 6,238 km and 17,240 km was 42%. Copper (Cu) concentration showed the most variability, ranging from 2.14 ppm (OP_2414) to 11.3 ppm (OP_2378), which may reflect differing levels of wear in copper-based bearings and other components. Similar copper levels were reported by Sejkorova et al.^20^ where exceeding the recommended 40,000 km oil change interval by 7,500 km resulted in Cu content of 7.5 ppm. Extending the interval by 17,500 km increased Cu to 9.2 ppm. For iron, however, more pronounced differences were noted. Exceeding the interval by 7,500 km led to 56 ppm Fe. Extending it by 17,500 km raised Fe to 68 ppm, representing an 18% increase in this element’s concentration. These findings align with the observed nonlinear relationship between oil degradation and wear metal accumulation.

Elemental composition analysis revealed changes in the concentration of performance-enhancing additives (Zn, P, S) in the used oils. In fresh oil, these values were: Zn—1376 ppm, P—960 ppm, and S—3806 ppm. In sample OP_2384, the largest decrease in additive content was observed: zinc (Zn): 22% decrease, phosphorus (P): 21% decrease, sulfur (S): 26% decrease, and calcium (Ca): 36% decrease. The chemical composition and additive levels directly impact the results of the four-ball test for lubricants. For instance, as shown in studies^21,22^, overbased calcium sulfonate additives demonstrated the ability to reduce wear scar diameter, indicating enhanced wear protection. In samples OP_2400 and OP_2420, Zn and P concentrations increased by 15–16%, likely due to varying degrees of oxidation. These fluctuations highlight the complex interactions between additive degradation and oxidation processes during oil use. Oil top-ups represent another potential explanation for these changes, as they may cause temporary increases in additive levels. However, precise data on the timing and quantities of these maintenance operations were not accessible. The actual values of anti-wear additives are presented in Table 7.

**Table 7:** Concentration of additives in the tested oils.

Operating condition	Sample code	Overall mileage [km]	Mileage on oil [km]	Zn [ppm]	P [ppm]	S [ppm]	Ca [ppm]	Mo [ppm]
				x̅ ± SD	x̅ ± SD	x̅ ± SD	x̅ ± SD	x̅ ± SD
New oil	OP	–	–	1376 ± 3	960 ± 31	3806 ± 14	4305 ± 15	379 ± 24
City	OP_2384	868,964	48,864	1077 ± 1	759 ± 26	2809 ± 34	2758 ± 20	417 ± 21
	OP_2414	704,454	66,238	1405 ± 1	907 ± 14	3594 ± 23	4614 ± 17	387 ± 11
	OP_2378	874,729	70,600	1544 ± 6	1001 ± 13	3616 ± 9	5072 ± 36	366 ± 9
	OP_2420	77,240	77,240	1579 ± 0	1116 ± 37	3570 ± 17	4850 ± 25	419 ± 11
	OP_2400	130,719	80,984	1576 ± 6	1082 ± 24	3504 ± 26	5073 ± 20	384 ± 22

x̅ arithmetic average, *SD* Standard deviation.

**Fig. 4:**
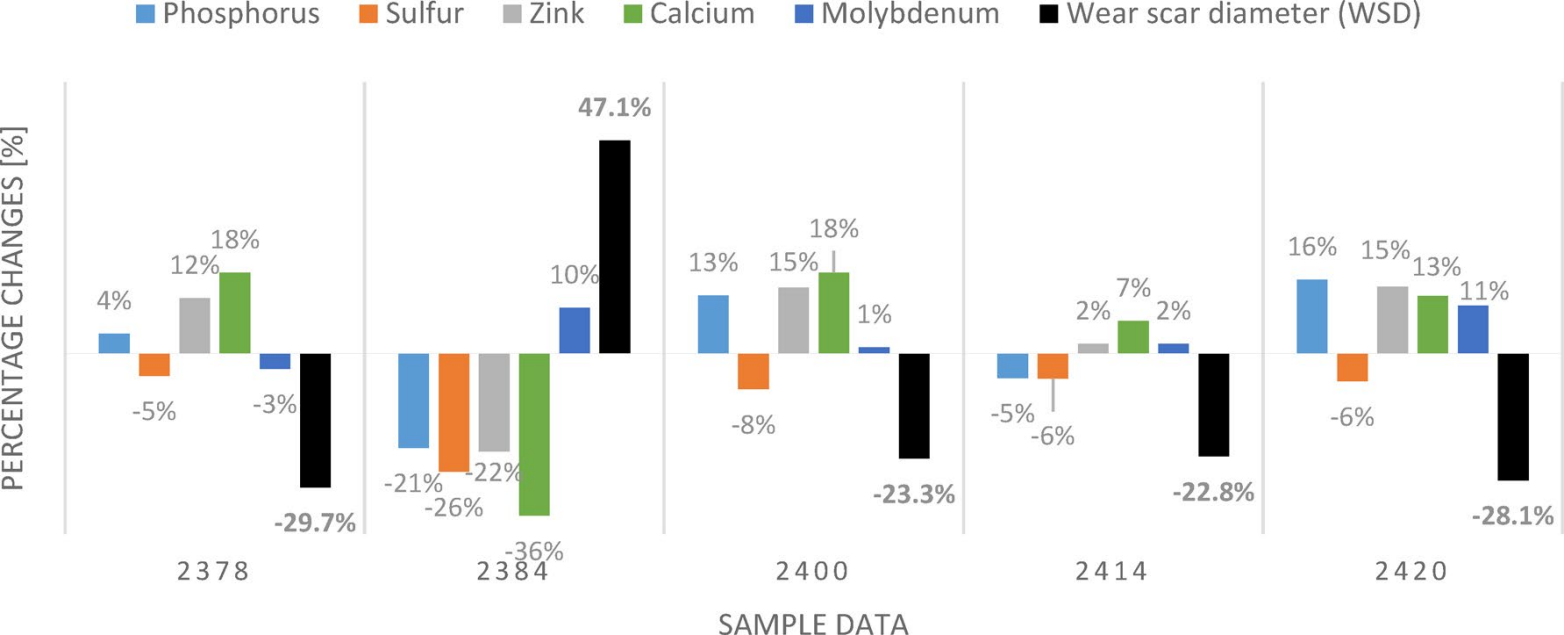
Percentage changes in additives and wear scar diameter in used engine oils in relevance to a new oil.

Figure 4 illustrates the percentage changes in the concentrations of elements associated with performance-enhancing additives in used engine oils compared to fresh oil, alongside the average wear scar diameters (WSD) of the balls. Based on the data presented in the figure, the impact of these percentage variations in elemental composition on the oils’ lubricating properties and their ability to protect against wear was evaluated.

Comparison of the five used oil samples revealed significant differences in the degree of degradation of performance-enhancing additives and the resulting changes in wear scar diameter (WSD). In the analyzed used oil samples, decreases were observed in the concentrations of elements such as zinc (Zn), phosphorus (P), and calcium (Ca), which play critical roles in anti-wear and detergent additives. Compared to fresh oil, the percentage differences for these elements ranged from tens to over forty percent, indicating chemical degradation of the additives during the operation of the city bus engines. The increase in WSD correlated with the reduction in additive element content. In sample 2384, which showed the largest decrease in zinc and phosphorus concentrations, the highest WSD values were recorded. This demonstrates a deterioration in the oil’s protective properties and an elevated risk of mechanical wear in engine components.

The concentration of additives in the oil should be noticeable, as observed in the case of bus OP_2384, but the increases in concentration were most likely due to the use of operational oil top-ups. According to data provided by the operator, the average top-up volume for this type of vehicle was approximately 1 L per 1000 km. However, these averages varied across different buses with varying operational mileage.

### Analysis of the physicochemical properties of oil—Eraspec Oil FTIR

**Fig. 5:**
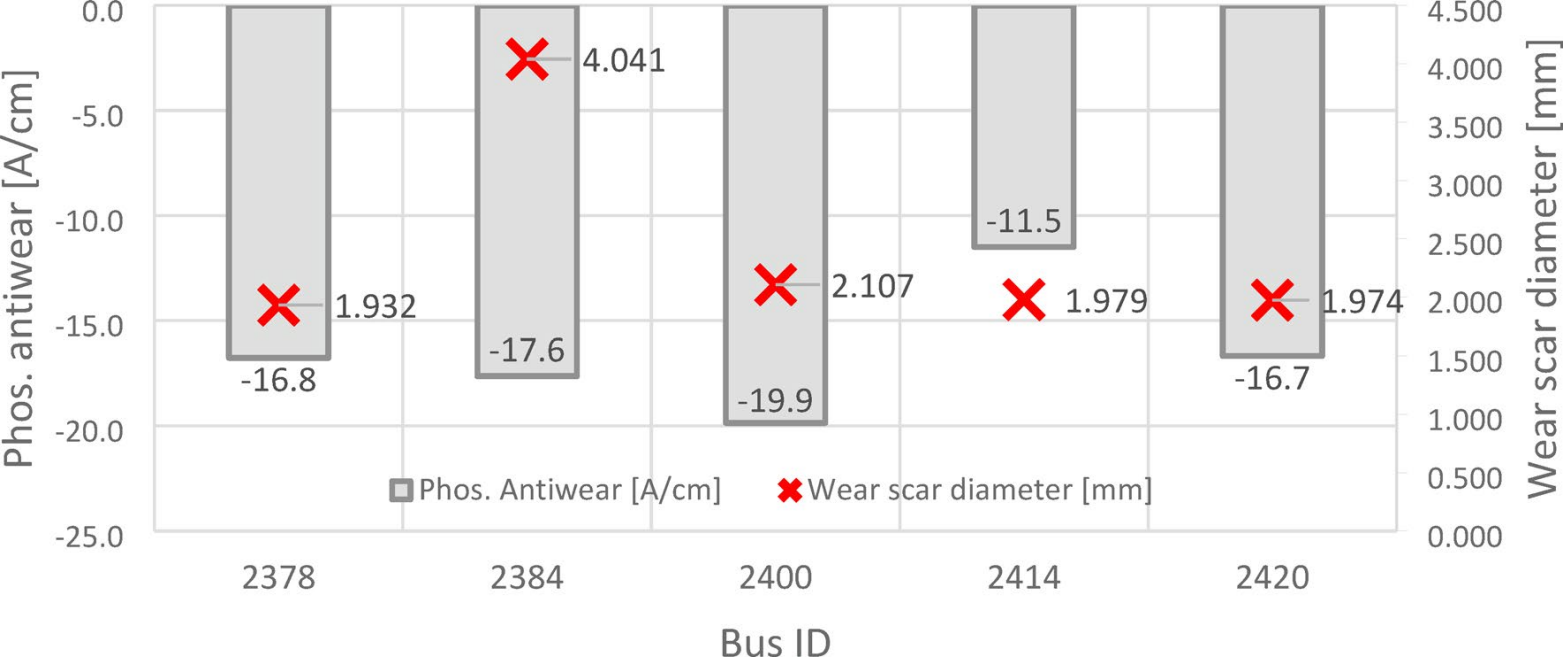
Depletion of Phos. antiwear additives and wear scar diameter in tested oils.

Monitoring the condition of oil requires tracking changes that occur during its use. For this purpose, the fresh oil spectrum (measured before use) is subtracted from the used oil spectrum. The resulting differential spectrum visualizes these changes. Positive signals in this spectrum indicate the presence of degradation products or contaminants, while negative signals reflect the depletion of additives. As shown in Fig. 5, spectral subtraction analysis-performed by the ASTM D7412 standard-was applied to evaluate phosphate anti-wear additives. These findings were then compared to the average wear scar diameter from tribological analysis, demonstrating the relationship between additive depletion and mechanical wear.

**Fig. 6:**
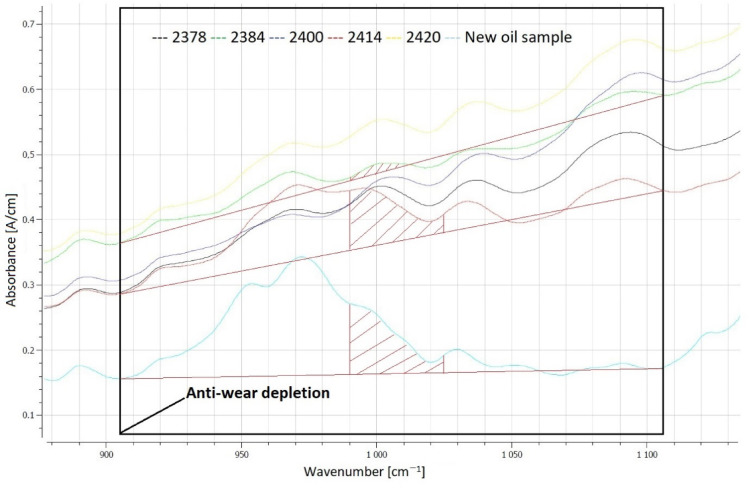
Evaluation of the area responsible for calculations of the remaining anti-wear additives.

The greatest depletion of anti-wear additives was observed in the oil sample labeled OP_2400 (a decrease of 19.9 [A/cm]), while the smallest depletion occurred in sample OP_2414 (a decrease of 11.5 [A/cm]). The largest reduction in phosphate anti-wear additives corresponded to the second-largest wear scar diameter of 2.107 mm. FTIR analysis revealed that in four out of the five post-change oil samples, the anti-wear additive content was 0%. Only in one case (sample OP_2414) were 80% of these additives detected. This highlights the relationship between additive depletion and wear severity under extended oil change intervals. FTIR spectra showing the area responsible for the calculation of the percentage of remaining anti-wear additives are shown in Fig. 6.

**Fig. 7:**
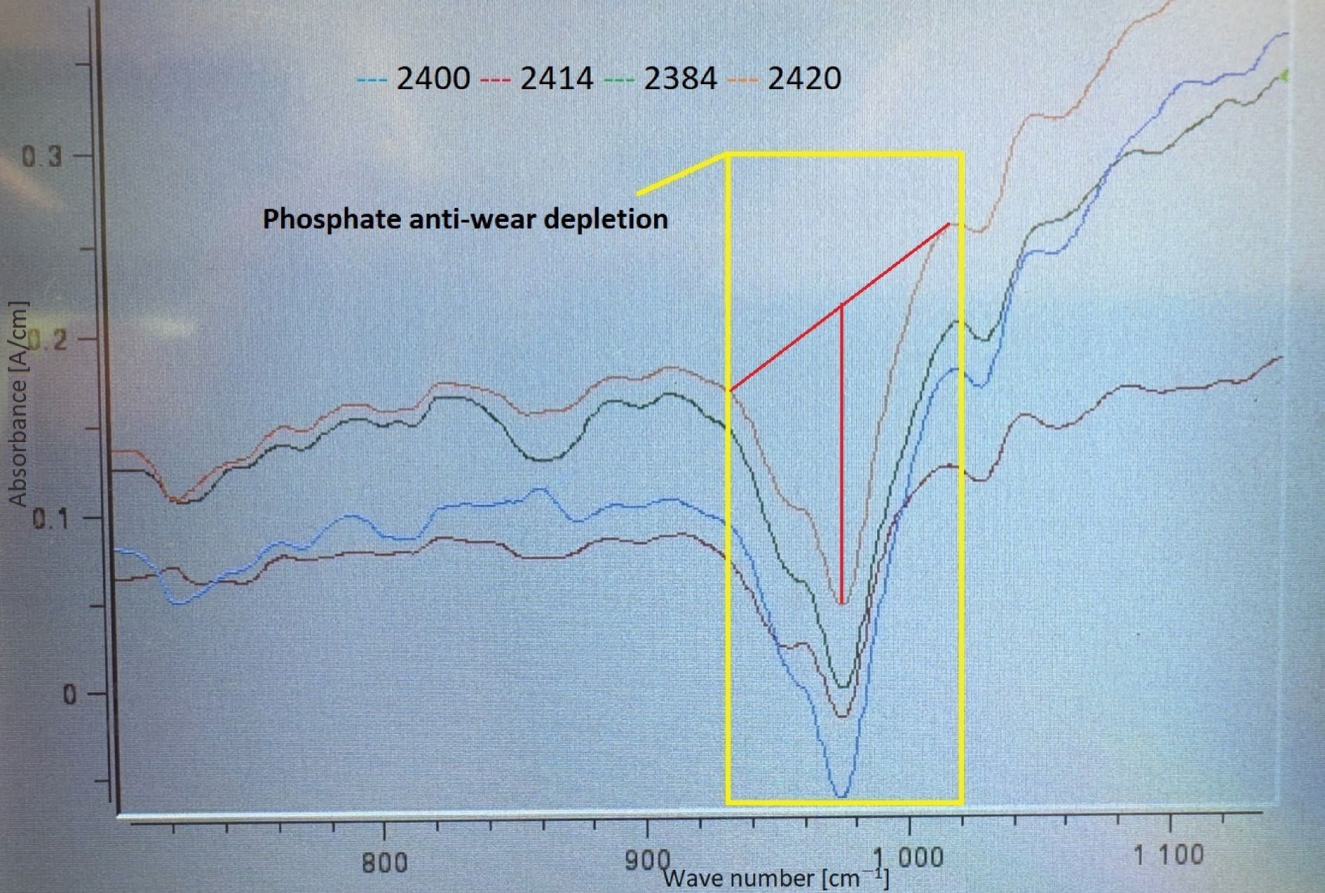
Evaluation of Phos. antiwear depletion is shown in red.

By subtracting the fresh oil spectrum from the used-oil spectrum, a so called difference spectrum is shown in Fig. 7. Negative signals result from the depletion of additives. Figure 7 highlights the evaluation of Phos. antiwear additives according to ASTM D7412.

**Fig. 8:**
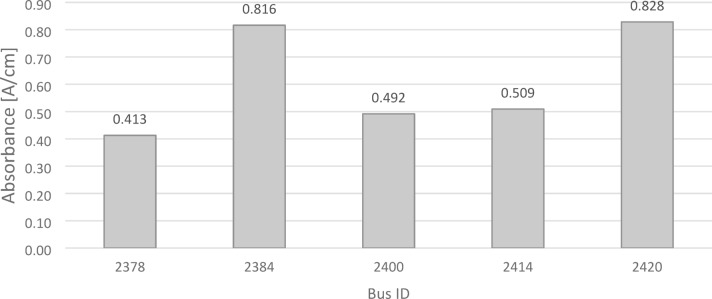
Soot in tested oils.

Analysis of the results presented in Fig. 8 revealed variation in soot content across the tested samples. The highest values were recorded in samples OP_2420 (0.828 A/cm) and OP_2384 (0.816 A/cm), while the lowest soot content was observed in sample OP_2378 (0.413 A/cm). Samples OP_2400 and OP_2414 showed intermediate values of 0.492 A/cm and 0.509 A/cm, respectively. The average soot content across all analyzed buses was 0.612 A/cm, with an average oil service mileage of 68,785 km. When compared to prior studies^23^, which analyzed differences in oil degradation between city and intercity buses: the current results are 48% higher than the average soot content reported for intercity buses, despite having a 56% lower average oil service mileage. For city buses in the previous study, soot levels were 71% higher than those in the current analysis, even though their average oil service mileage was 57% lower. These findings highlight the accelerated soot accumulation in urban bus engines under stop-and-go driving conditions, even at reduced mileage intervals^23^.

Comparing the data from Fig. 8 with the information presented in Fig. 5, an interesting relationship between soot content and the depletion of anti-wear additives was identified. Sample OP_2400, which exhibited the highest depletion of anti-wear additives (a decrease of 19.9 A/cm), showed a relatively low soot content (0.492 A/cm). In contrast, sample OP_2414, with the smallest depletion of anti-wear additives (a decrease of 11.5 A/cm) and 80% retention of these additives, displayed a comparable soot content (0.509 A/cm). This suggests that additive depletion does not always correlate linearly with soot accumulation, highlighting the complex interplay between additive degradation and contaminant formation in engine oils.

**Fig. 9:**
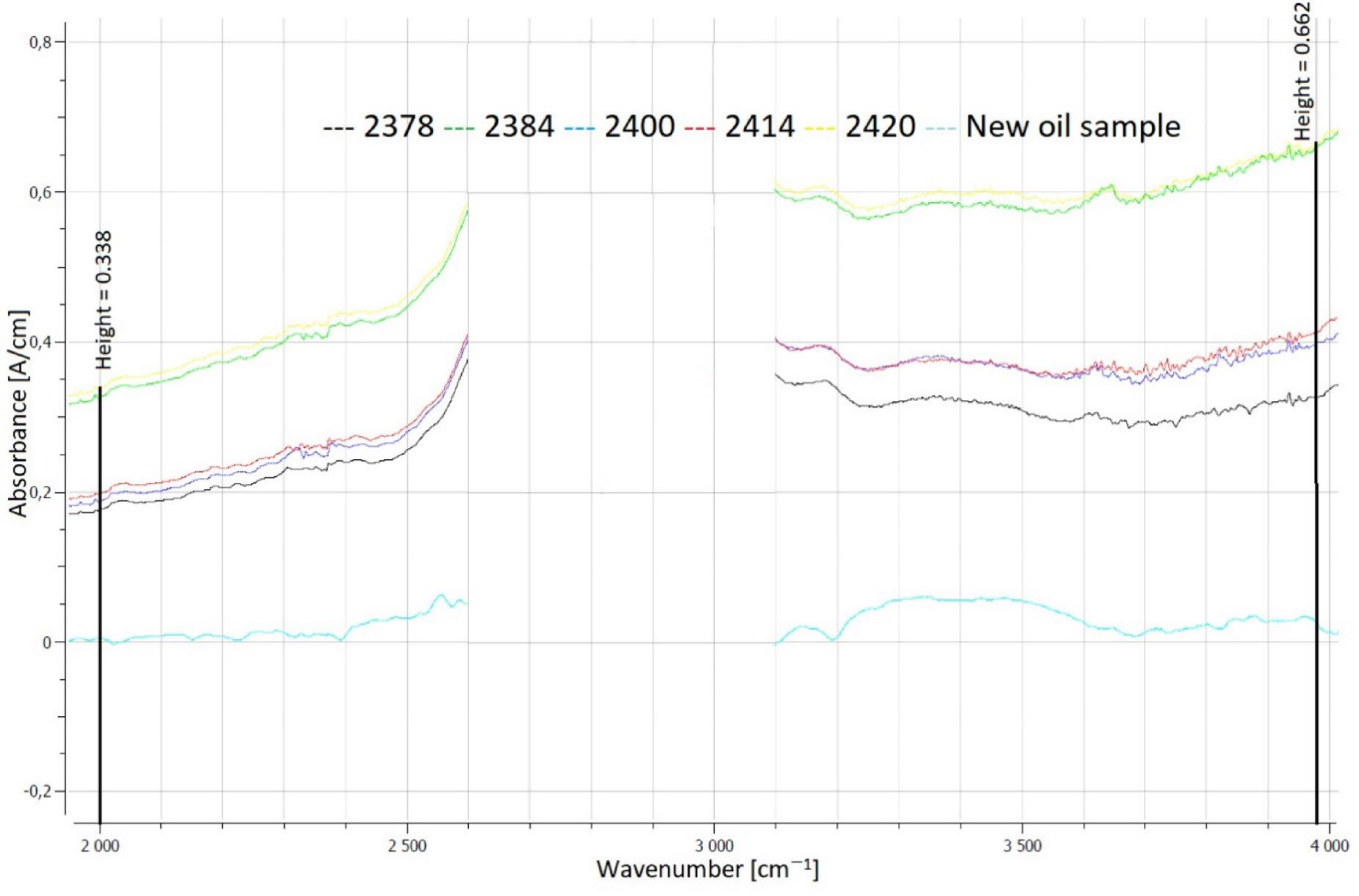
Evaluation of the soot content in oil.

The results of the study indicated significant differences in soot content among the samples. These variations could be attributed to differences in the engines’ total operational mileage or oil change schedules. A particularly notable case was sample OP_2414, which, despite having similar soot content to sample OP_2400, exhibited significantly better retention of anti-wear additives. FTIR analysis to determine soot content was conducted at two designated measurement points. Distinct absorption bands indicative of soot contamination were identified at wavelengths of approximately 2000 cm^−1^, and 3980 cm^−1^. Figure 9 demonstrates the differences among the analyzed oils at these specified points.

Based on the analysis of Fig. 8, it was concluded that soot content alone is not a sufficient indicator of engine oil condition. Other parameters, such as the degree of degradation of performance-enhancing additives, must also be considered. The highest soot content in sample OP_2420 contributed to increased mechanical wear of engine components, as soot particles act as abrasive agents. This finding was corroborated by iron content analysis, which also revealed the highest iron concentration (Fe: 132 ppm) in this sample compared to all others analyzed. The combined data underscores how soot accumulation and additive degradation synergistically accelerate engine wear under extended oil use.

The conducted analysis confirmed the importance of a comprehensive approach to assessing engine oil condition, which must account for both soot content and the degree of degradation of performance-enhancing additives, to ensure optimal operating conditions for city bus engines.

### Statistical analysis results

#### ANOVA analysis and an outlier identification

After verifying the assumptions, an ANOVA analysis was conducted. For the limit seizure pressure, the p-value was < 0.05, leading to the rejection of the null hypothesis that all variables exhibit the same resistance to wear in the tested contact under sliding friction conditions. This confirmed statistically significant differences between at least one pair of buses. The next step involved identifying which specific bus pairs differed significantly. The results of the ANOVA analysis are presented in consolidated Table 8.

**Table 8:** The mean values, standard deviations, and *p values after conducting specific laboratory tests of used engine oils.

	Bus ID		2378 {1}	2384 {2}	2400 {3}	2414 {4}	2420 {5}			
	*N*		3	3	3	3	3			
4-ball test	Wear scar diameter [mm]	x̅ (SD)	1.932 (0.066)	4.041 (0.053)	2.107 (0.039)	1.980 (0.012)	1.974 (0.057)			
Elemental analysis	Iron [Fe]	x̅ (SD)	62.75 (0.83)	76.14 (0.06)	96.37 (0.81)	75.87 (0.58)	131.7 (0.10)			
	Copper [Cu]		11.3 (0.10)	4.48 (0.09)	9.30 (0.02)	2.14 (0.14)	5.26 (0.17)			
	Molybdenum [Mo]		366 (9)	417 (21)	384 (22)	387 (11)	419 (11)			
	Zinc [Zn]		1544 (6)	1077 (1)	1576 (6)	1405 (1)	1579 (0)			
	Phosphorus [P]		1001 (13)	759 (26)	1082 (24)	907 (14)	1116 (37)			
	Sulfur [S]		3616 (9)	2809 (34)	3504 (26)	3594 (23)	3570 (17)			
FTIR	Phos. antiwear [A/cm]	x̅ (SD)	− 16.76 (0.03)	− 17.63 (0.06)	− 19.85 (0.05)	− 11.50 (0.05)	− 16.67 (0.05)			
	Soot [A/cm]		0.413 (0.00)	0.816 (0.01)	0.492 (0.01)	0.509 (0.00)	0.828 (0.01)			

	{5}–{4}	{5}–{3}	{5}–{2}	{5}–{1}	{4}–{3}	{4}–{2}	{4}–{1}	{3}–{2}	{3}–{1}	{2}–{1}
*p-*value	0.999	0.504	**< 0.001**	0.825	0.059	**< 0.001**	0.774	**< 0.001**	**0.010**	**< 0.001**
*p-*value	**< 0.001**	**< 0.001**	**< 0.001**	**< 0.001**	**< 0.001**	0.996	**< 0.001**	**< 0.001**	**< 0.001**	**< 0.001**
	**< 0.001**	**< 0.001**	**< 0.001**	**< 0.001**	**< 0.001**	**< 0.001**	**< 0.001**	**< 0.001**	**< 0.001**	**< 0.001**
	0.170	0.126	0.999	**0.015**	0.999	0.228	0.535	0.170	0.650	**0.021**
	**< 0.001**	0.851	**< 0.001**	**< 0.001**	**< 0.001**	**< 0.001**	**< 0.001**	**< 0.001**	**< 0.001**	**< 0.001**
	**< 0.001**	0.482	**< 0.001**	**0.001**	**< 0.001**	**< 0.001**	**0.006**	**< 0.001**	**0.015**	**< 0.001**
	0.723	**0.037**	**< 0.001**	0.197	**0.005**	**< 0.001**	0.787	**< 0.001**	**0.001**	**< 0.001**
*p-*value	**< 0.001**	**< 0.001**	**< 0.001**	0.183	**< 0.001**	**< 0.001**	**< 0.001**	**< 0.001**	**< 0.001**	**< 0.001**
	**< 0.001**	**< 0.001**	0.452	**< 0.001**	0.154	**< 0.001**	**< 0.001**	**< 0.001**	**< 0.001**	**< 0.001**

*N* number of samples tested*, x̅* arithmetic average, *SD* standard deviation, *p value resulting from the application of Tukey’s post-hoc test.

Oil groups (e.g., {5}–{4}) represent a comparison between two distinct oil samples. Analyzing the results for individual samples helps identify similar oils or distinguish those that differ.

Significant values are in bold.

Results with a p-value below this threshold were considered statistically significant and marked in bold. Those with a p-value below 0.01 were classified as highly statistically significant, indicated by bold text with underlining.

**Fig. 10:**
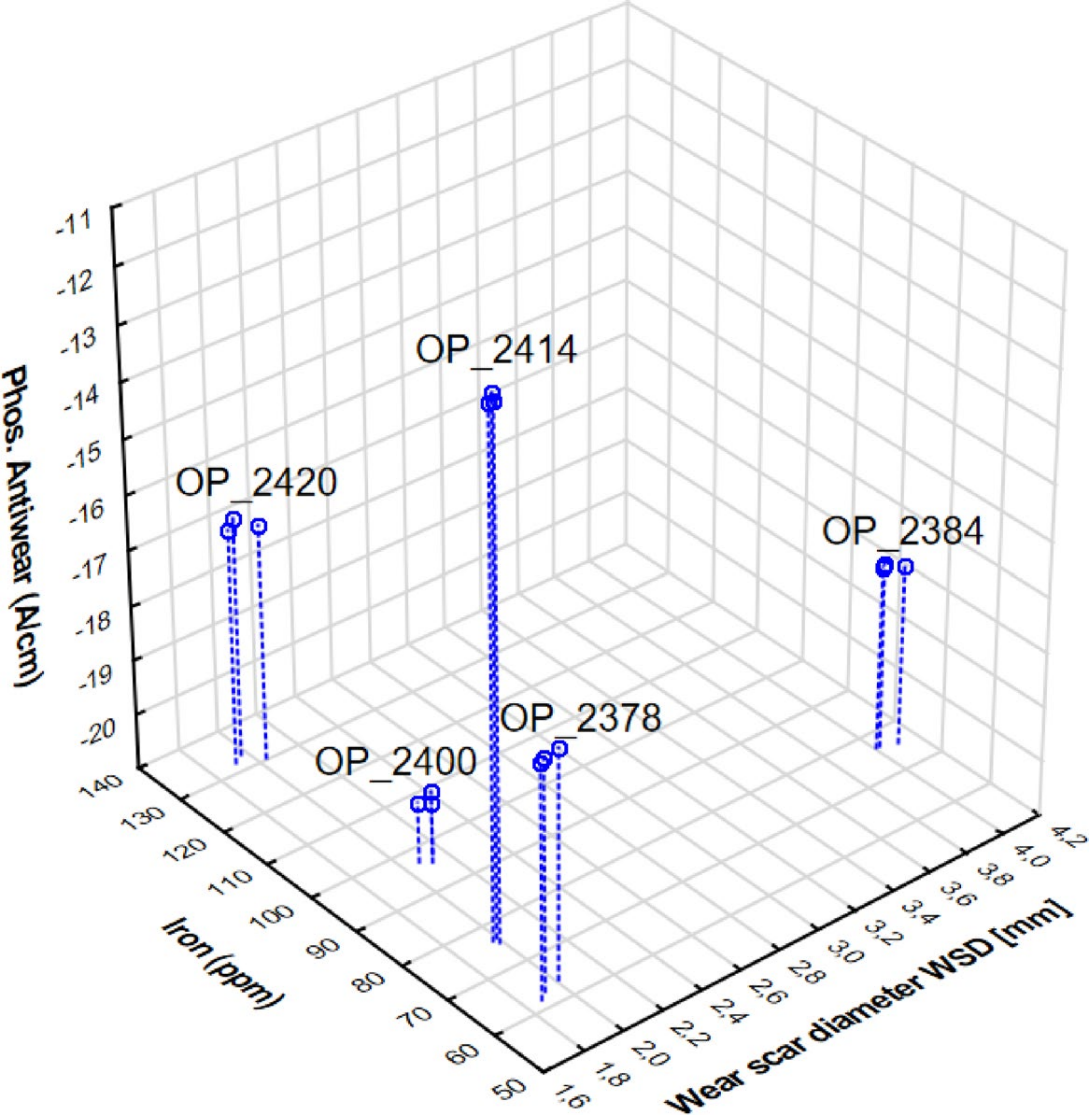
Scatter plot of iron content, wear scar diameter and depletion of anti-wear additives.

After analyzing the individual variables presented in Table 8, it was found that, based on the four-ball test results, all of the tested oils- except for the oil from bus OP_2384- showed similar (homogeneous) wear scar diameters. This indicates that the oil from bus OP_2384 stands out as an outlier. The differences between the oils tested are visually represented in Fig. 10.

In investigating the cause of the outlier average wear scar diameter observed in the tribological analysis of bus OP_2384, the vehicle’s service history was carefully reviewed. Two months after an oil change performed at 874,803 km, a failure was identified that required the engine to be disassembled and repaired. Earlier service records revealed several potential factors that could have contributed to this failure. The repair history showed recurring problems with the turbocharger dating back to May 2023, as well as issues with improper operation of the fuel injectors. Additionally, there were multiple cooling system problems, including a “broken water hose in the engine cylinder head” in July 2023, which suggested possible cylinder head issues. Numerous cooling system repairs were carried out in June and July 2024, indicating ongoing difficulties in maintaining the engine’s proper operating temperature. Between May and December 2023, at least nine interventions were made to address oil leaks. The high frequency of these recurring issues strongly suggests the presence of a serious underlying structural problem in the engine.

In summary, the simultaneous occurrence of problems with the oil system, cooling system, and electronic control system represented a clear accumulation of risk factors for a major failure. The primary cause of the breakdown was most likely a combination of several technical issues-turbocharger damage, oil system sealing problems, and improper operation of the cooling system. These factors can disrupt the normal oil circulation in the engine, alter pressure in the lubrication system, cause uneven lubrication of friction components, and result in selective action of performance additives. All of these factors can directly lead to a deterioration of the oil’s anti-wear properties, which was reflected in the high WSD (wear scar diameter) value.

#### Pearson correlation coefficient and regression analysis

**Fig. 11:**
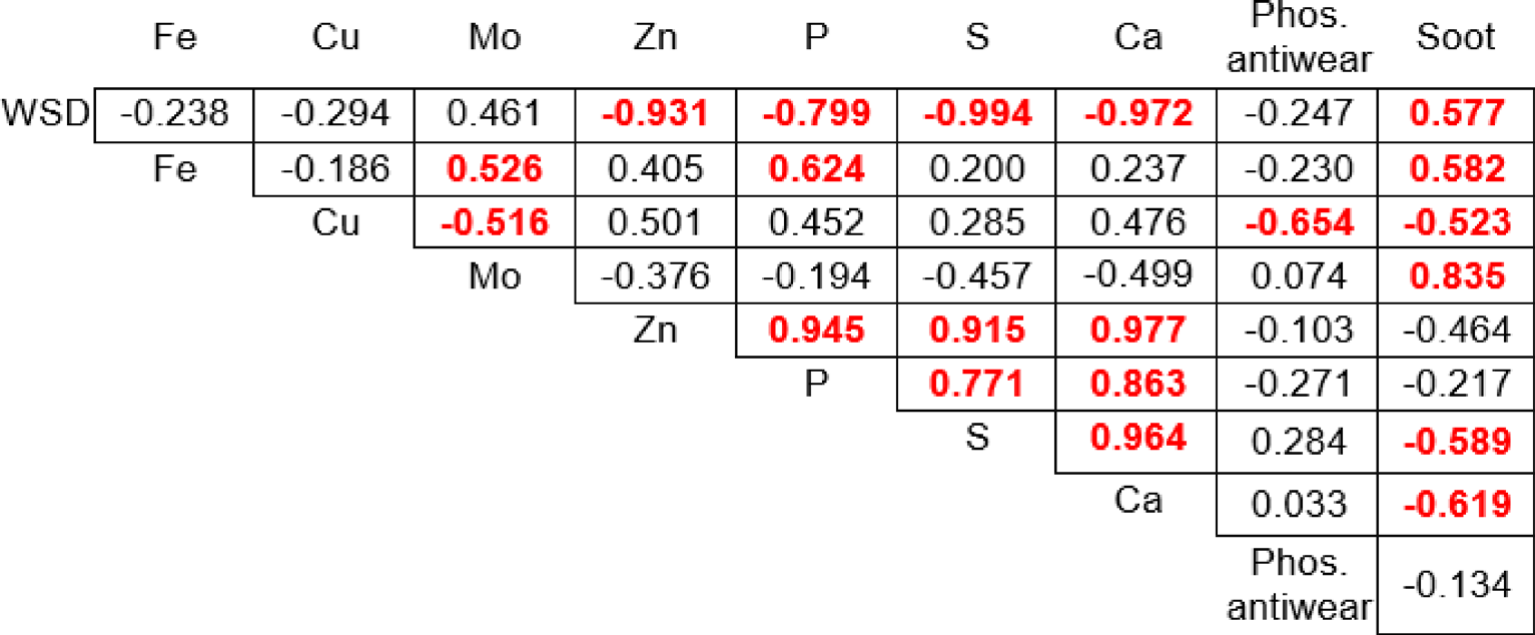
Correlation between the selected indicators, those significant at the 5% level are red coloured.

The correlation matrix shown in Fig. 11 provides insight into the interrelationships between various parameters of used engine oils from all the tested city buses. Statistically significant results are marked in red.

The analysis first focused on the correlation between the depletion of phosphate anti-wear additives (Phos. antiwear) and wear protection parameter.

For the first variable, wear scar diameter (WSD), a weak negative correlation with Phos. antiwear was observed (r =  − 0.247). This indicates that as the level of anti-wear additives decreases, WSD slightly increases, reflecting reduced wear protection.

Next, regression analysis was performed to determine the direct correlation between each individual oil property and wear scar diameter. The resulting equation is as follows:

3 $$Wear \;scar\; diameter \;WSD={b}_{0}+{b}_{1}x$$

where, *b*_*0*_ and *b*_*1*_ are the regression function’s structural parameters, and *x* is the independent variable.

For wear protection parameters such as wear scar diameter (WSD), a strong negative correlation was observed with zinc (r =  − 0.931) and phosphorus (r =  − 0.799) content. This suggests that lower levels of these elements in the oil samples are associated with increased wear scar diameter WSD values. However, given the small sample size, these associations should be interpreted with caution, as they may not hold in larger datasets. Subsequently, regression analysis was performed to determine the direct correlation between zinc and phosphorus levels and wear scar diameter. This resulted in the following equations:

4 $$Wear \; scar\; diameter \;WSD=7.2922+\left(-0.0050\times Phosphorus\right)$$

5 $$Wear \;scar \;diameter \;WSD=8.1703+\left(-0.0040\times Zinc\right)$$

**Fig. 12:**
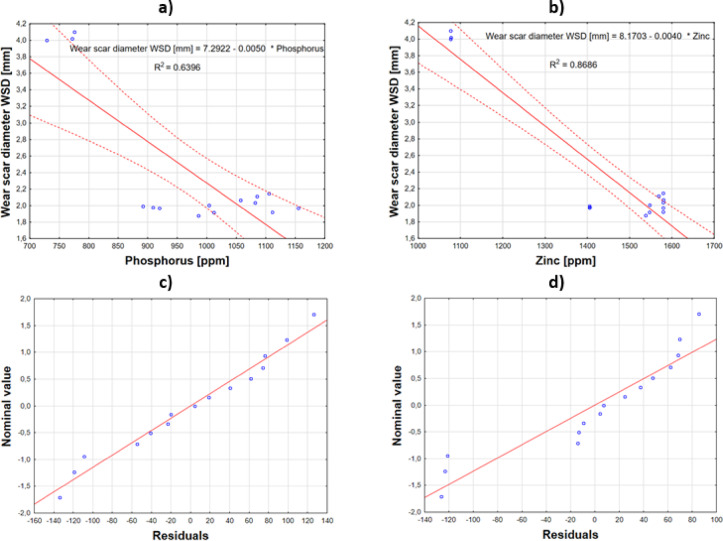
Correlations between depletion of (**a**) phosphorus and (**b**) zinc on wear scar diameter and (**c**) and (**d**) are corresponding normality plots of residuals from regression analysis correlating WSD vs phosphorus and zinc.

Correlation plots showing the relationship between the depletion of anti-wear additives, such as zinc dialkyldithiophosphates (ZDDP), and the wear scar diameter are presented in Fig. 12.

The normality plots of residuals (Fig. 12c and d) show that the points align closely along the straight line (or near it) without systematic deviations. This suggests no grounds to reject the assumption of residual normality and, consequently, supports the linearity of the model.

A positive correlation was observed between soot levels [A/cm] (r = 0.577) and wear scar diameter (WSD), suggesting that higher soot content in the oil samples is associated with increased wear.

The strongest associations were found with sulfur (r =  − 0.9946) and calcium (r =  − 0.9724) levels, where lower concentrations of these elements corresponded to larger average WSD values. Similarly as with phosphorus and zinc, it should be noted that these correlations are based on a small sample size and may not be representative of larger datasets. Subsequently, regression analysis was conducted to determine the direct correlation between sulfur/calcium levels and wear scar diameter. The resulting equations are as follows:

6 $$Wear \;scar \;diameter \;WSD \left[mm\right]=11.471+\left(-0.0027\times Sulphur\right)$$

7 $$Wear\; scar\; diameter \;WSD \left[mm\right]=6.485+\left(-0.0009\times Calcium\right)$$

**Fig. 13:**
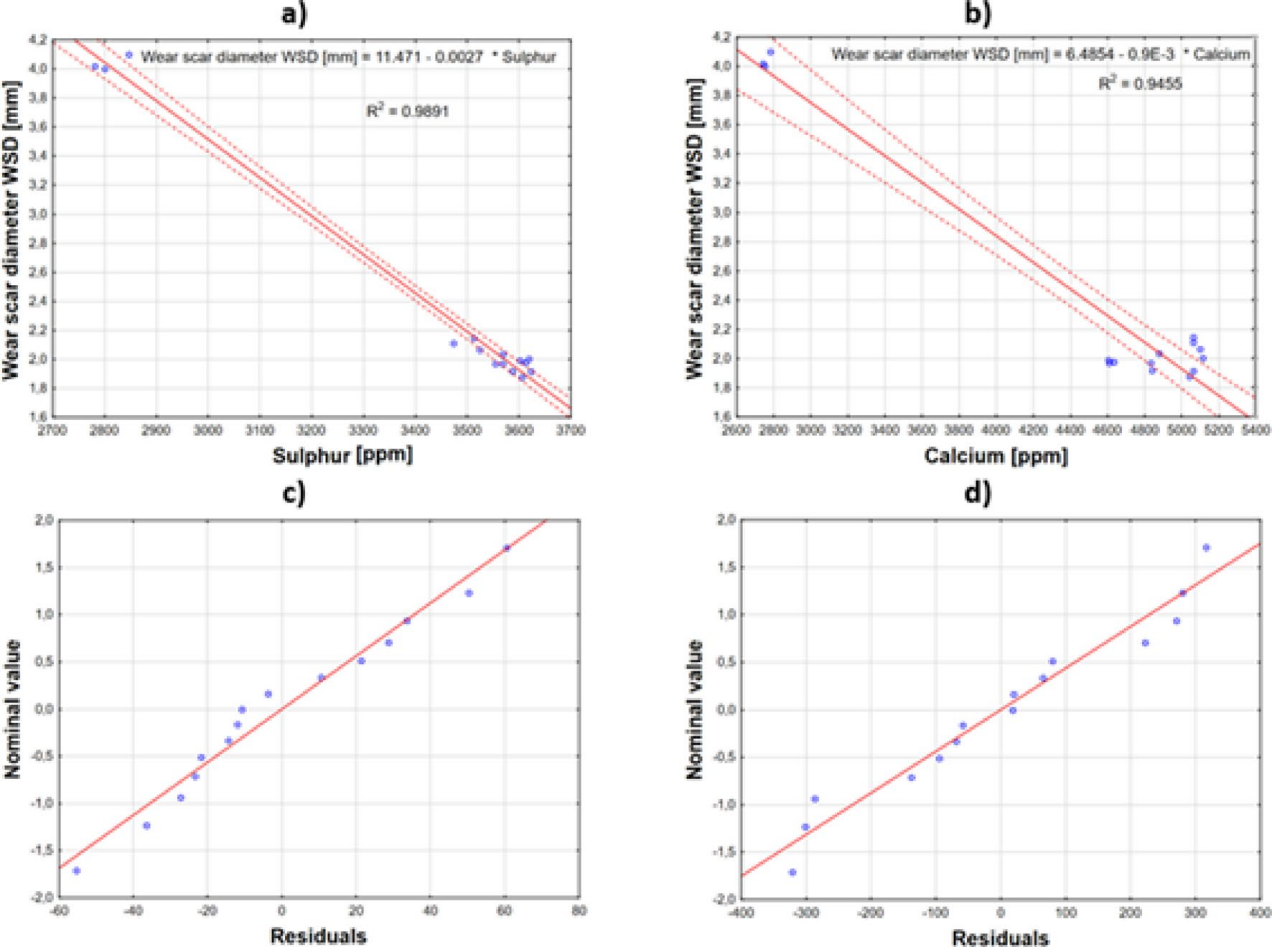
Wear scar diameter vs best correlating variables, (**a**) sulphur and (**b**) calcium and (**c**) and (**d**) are corresponding normality plots of residuals from regression analysis correlating WSD with sulphur and calcium.

To visualize this correlation, the combined independent variables are presented on the plot relative to the wear scar diameter using Eqs. 6 and 7 (Fig. 13a and b). The coefficient of determination R^2^ for sulfur was 0.9891, and for calcium, 0.9455. From the residual normality plots, it can be observed that the position of the points on the plot relative to the overlaid lines suggests no grounds to reject the assumption regarding the model’s form (Fig. 12c and d). The p-value for the regression coefficient for both sulfur and calcium was *p* < 0.005, and the adjusted coefficient of determination R^2^ was 0.9883 for sulfur and 0.9413 for calcium, with confidence intervals of 0.95.

The observed strong negative correlation between sulfur content and wear scar diameter (r = − 0.9946) is confirmed in the work of Spikes^24^, who described in detail the mechanisms of sulfur-containing additives in engine oils. As shown in our study, an increase in sulfur content leads to a significant reduction in wear scar diameter according to Eq. (6), which aligns with the results obtained by Martini et al.^25^. These authors demonstrated that sulfur-containing additives form mechanically strong reaction layers on friction surfaces.

The correlation analysis between calcium content and wear scar diameter (WSD) (r = − 0.9724) suggests that calcium may be associated with the tribological properties of the tested oils. However, this observed relationship is based on a limited sample size and should be interpreted with caution. Our results correspond with the work of Ratoi et al.^26^, who showed that calcium-based detergents not only neutralize acidic combustion products but also participate in forming protective layers on metal surfaces, thereby reducing engine component wear.

The regression model describing the relationship between calcium content and WSD (Eq. 7) features a slightly lower but still high coefficient of determination (R^2^ = 0.9455), confirming the observations of Uy et al.^27^. In their work, these authors stated that a decrease in calcium content in engine oil by 1,000 ppm leads to an increase in wear scar diameter by approximately 0.9 mm, indicating a linear relationship between these parameters.

Rudnick^28^, in his comprehensive monograph on engine oil additives, notes that the occurrence of strong negative correlations between sulfur/calcium content and wear parameters is typical for modern engine oils, and coefficient of determination values above 0.90 indicate a good regression model fit. Our study confirms these observations, achieving R^2^ values of 0.9891 for sulfur and 0.9455 for calcium. Costa and Spikes^29^ obtained similar coefficients of determination in their studies (R^2^ = 0.97 for sulfur and R^2^ = 0.93 for calcium), which confirms the reliability of our results. The models are statistically reliable, but limitations related to the small sample size require further analysis.

Next, attention was focused on the contamination and degradation of the oil. Looking at soot levels, a weak negative correlation with Phos. antiwear (e.g., Soot [A/cm] r =  − 0.134) suggested a limited direct impact on additive depletion. A moderate positive correlation was observed between soot levels [A/cm] (r = 0.577) and wear scar diameter (WSD), indicating that higher soot content in the oil samples is associated with increased wear.

#### Robust method analysis-Spearman’s rank correlation

In order to obtain a more complete picture of the dependencies in the data and to assess the impact of outliers regarding the OP_2384 bus, it was decided to supplement the analyses with robust methods, such as Spearman’s rank correlation. The results and differences between Pearson correlation coefficient are presented in Table 9. Statistically significant results from robust method are marked in red.

**Table 9:** Spearman’s rank correlation results and comparison between used methods.

Pair of variables	Spearman’s rank correlation			Comparison with Pearson correlation	
	Spearman’s R	t(N-2)	*p*-value	Differences between methods	Conclusions
Wear scar diameter WSD & Phosphorus	− 0.354	− 1.36	0.20	Large (∆R = 0.445)	Outliers inflate Pearson
Wear scar diameter WSD & Zinc	− 0.264	− 0.98	0.34	Huge (∆R = 0.667)	Extreme distortion of Pearson
Wear scar diameter WSD & Sulfur	**− 0.810**	**− 4.97**	**< 0.05**	Small (∆R = 0.184)	Consistent confirmation of a strong association
Wear scar diameter WSD & Calcium	− 0.304	− 1.15	0.27	Huge (∆R = 0.668)	Pearson completely distorted
Wear scar diameter WSD & Soot	0.304	1.15	0.27	Moderate (∆R = 0.273)	Outliers affect Pearson
Wear scar diameter WSD & Phos. antiwear	**− 0.617**	**− 2.82**	**< 0.05**	Large (∆R = 0.370)	Only Spearman shows significance. Monotonic but nonlinear relationship

Significant values are in bold.

The comparative analysis between Spearman’s rank correlation and Pearson’s correlation revealed significant discrepancies across most variable pairs, indicating substantial outlier influence in the dataset. For most variables (phosphorus, zinc, calcium, soot), Pearson correlation showed artificially inflated, statistically significant relationships, while Spearman’s robust method indicated these associations were actually weak or non-significant. Only sulfur demonstrated strong, consistent negative correlations in both methods, confirming a genuine, robust relationship with wear scar diameter. Phos. antiwear additives showed significant correlation only with Spearman’s method, suggesting a monotonic but non-linear relationship that Pearson failed to detect appropriately.

In summary, the findings from the regression analysis should be interpreted with considerable caution due to the limited number of observations. The data suggest that lower levels of Phos. antiwear additives are associated with increased wear scar diameter (WSD), indicating a possible relationship with tribological performance. Soot accumulation appeared to be associated with higher wear, although its direct relationship with additive depletion was limited. Additionally, higher concentrations of iron and copper in used oils were observed alongside lower additive levels, highlighting an association between metal content and additive depletion. These results underscore the potential value of timely oil replacement in relation to engine component wear, but further studies with larger sample sizes are needed to confirm these patterns.

### Analysis of the impact of exceeding the engine oil change interval

**Fig. 14:**
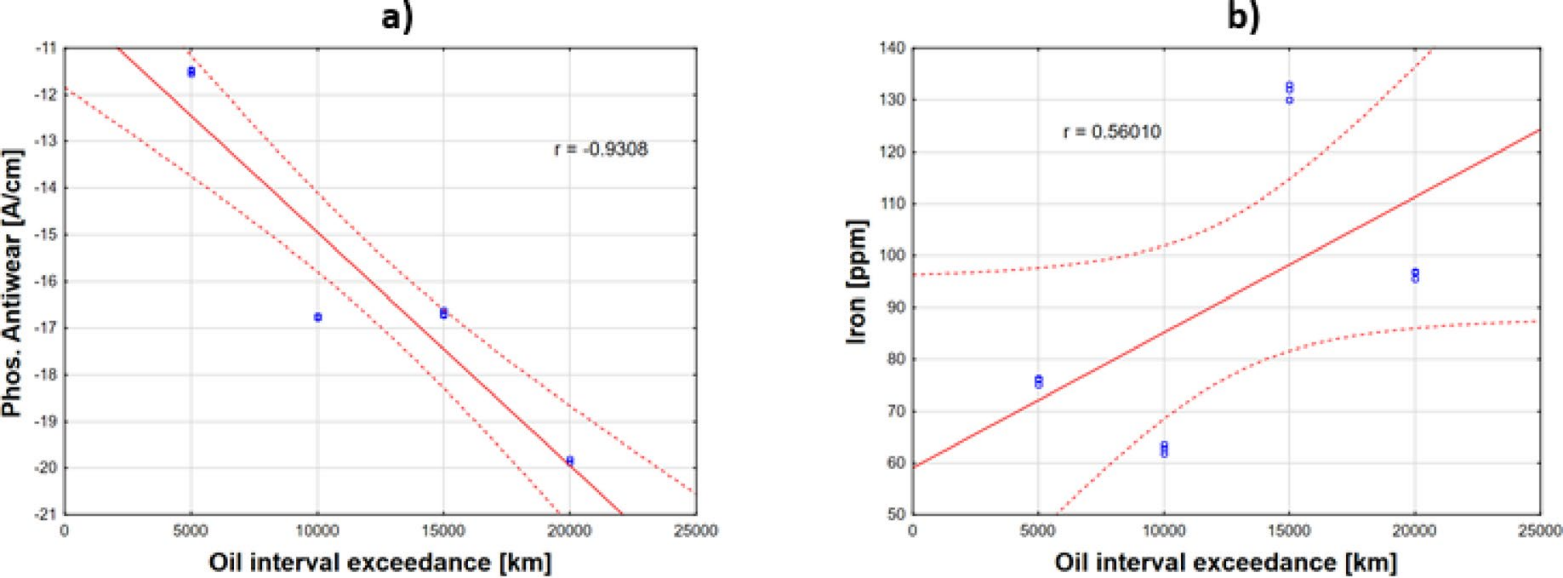
Effects of oil interval exceedances on depletion of (**a**) antiwear additives and (**b**) iron content.

**Fig. 15:**
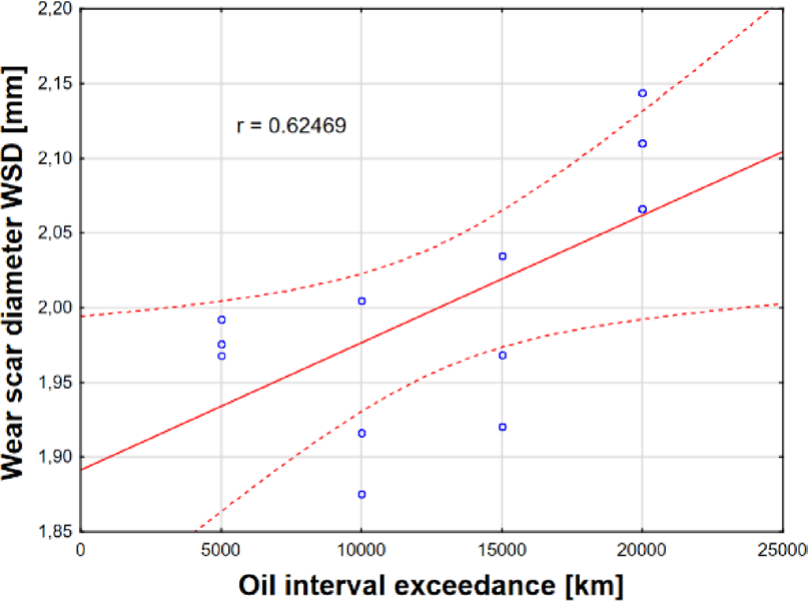
The impact of exceeding the oil interval on the amount of wear of engine components.

The following Figs. 14 and 15 present an analysis of the impact of exceeding the recommended oil change interval on the depletion of Phos. antiwear additives, the increase in wear metal content, and the intensification of the wear scar diameter (WSD) of the balls.

Figure 14a reveals a strong negative correlation (r =  − 0.9308), indicating that exceeding the recommended oil change interval leads to a decline in Phos. antiwear additive content. When the interval is exceeded by 5,000 km, additive levels decrease but still provide minimal wear protection, with an average iron content of 76 ppm (Fig. 14b).

A strong positive correlation (r = 0.5601) was observed between extended oil intervals and rising iron content. After 10,000 km beyond the interval, additive depletion becomes more pronounced, showing a 46% increase compared to the 5,000 km overage period. However, iron content only rose to 63 ppm during this phase.

Accelerated wear processes became evident at 15,000 km beyond the interval, where iron content surged to 132 ppm, signaling severe engine component wear. Exceeding the interval by 15,000–20,000 km raises the risk of mechanical failures (e.g., seizure). At 20,000 km overage, Phos. antiwear additive depletion was 73% higher than at 5,000 km overage, highlighting the compounding impact of delayed oil changes.

The degradation of Phos. antiwear additives was observed to be nonlinear, with each subsequent interval exceedance accelerating their depletion rate. Extending the oil change interval caused a disproportionate increase in engine component wear, which may lead to high operational costs and a heightened risk of mechanical failure^10,11^. When comparing our results with those obtained in previous field studies by other researchers, according to Dörr et al.^12^ ZDDP compounds degrade rapidly, with intermediate products forming and depleting within the first 6,000 km. The presence of intact ZDDP correlates with lower wear rates, while its depletion leads to increased wear. Raposo et al.^5^ in their field studies based on Fe degradation trends, determined that there is a basis for extending oil change intervals by 25%, from 20,000 km to 25,000 km. Valis et al.^30^ introduced predictive modeling of engine failures also based on wear metals, focusing on the linear progression of Fe and Pb. The threshold limit for Fe was set at 90 ppm.

Analyzing the wear scar diameter (WSD) of the balls measure of engine component wear, plotted against oil change interval exceedance (Fig. 15), a strong positive correlation (r = 0.6246) was observed, indicating intensified wear processes as the recommended oil change interval is exceeded. Exceeding the oil change interval leads to a systematic deterioration of the oil’s lubricating properties. Furthermore, additive degradation and engine component wear accelerate as the deviation from the recommended interval increases^17^.

**Fig. 16:**
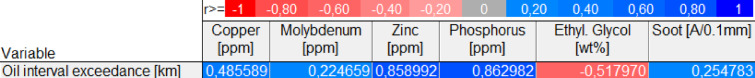
Correlation matrix of oil interval exceedance vs other variables.

Figure 16 presents the correlations showing the impact of exceeding the manufacturer’s recommended oil change interval on the other variables studied.

Notably, a strong positive correlation was observed between increasing zinc and phosphorus content and extended oil change interval exceedance. In general, ZDDP (zinc dialkyldithiophosphate) degrades over time, leading to the formation of various zinc-containing compounds^12,13^. Literature studies have shown that used engine oils contain significant amounts of zinc, with concentrations varying widely^14–16^. While zinc concentration in used oils does not necessarily increase linearly with mileage, it may depend on ZDDP depletion and the formation of wear particles^31^.

In summary, analysis of the results indicates that exceeding the recommended oil change interval by more than 10,000 km increases the risk to engine longevity. When the interval is exceeded by more than 15,000–20,000 km, the likelihood of serious engine failures rises sharply. Therefore, it is strongly recommended to follow the manufacturer’s recommended oil change interval (60,000 km) to minimize the risk of damage and keep operating costs under control.

Routine use of oil analysis methods, such as FTIR, viscosity analysis, or elemental analysis, can be technically justified, especially in environments where vehicle reliability is critical. Regular monitoring of oil condition allows for early detection of abnormalities, which can prevent serious failures and the associated repair costs and downtime.

From an economic perspective, the costs of implementing such a system must be compared with the potential savings resulting from avoiding costly repairs and operational fleet losses. Our study showed that these methods can effectively identify problems before they become critical, potentially reducing operating costs.

However, the economic efficiency of these methods depends on the scale of their application and integration with other maintenance procedures. We propose further analyses to determine optimal monitoring intervals and the costs and benefits of routine use of these methods in a broader operational context.

## Conclusions

The research results provided valuable information for bus fleet operators, enabling more precise determination of engine oil change intervals, which in turn extends engine lifespan and reduces operating costs.

The case of bus OP_2384 illustrates how severe engine damage can affect the tribological properties of engine oil, further accelerating degradation processes and potentially leading to complete engine seizure if the issue is not identified and addressed in time.

The tribological and physicochemical analyses of engine oils used in city buses allowed for the formulation of the following conclusions:

1. The tribological analysis revealed an association between engine oil use and a reduction in anti-wear properties, with the extent of this reduction not appearing to be linearly related to mileage. Fresh oil samples showed the highest seizure point (2800 N), whereas used samples exhibited lower values, down to 950 N
2. A strong negative Pearson’s correlation (r = − 0.9946) was observed between sulfur content and wear scar diameter (WSD) in the analyzed samples, suggesting a potential relationship between sulfur compounds and the oil’s lubricating characteristics. However, given the small sample size, this association may not persist in larger datasets.
3. Spearman’s correlation proved more reliable for this dataset due to outlier presence, with sulfur being the only parameter showing consistently strong associations with engine wear across both analytical approaches.
4. The sample with the greatest oil change interval exceedance (OP_2420) had the highest iron concentration (131.67 ppm), which may be associated with more pronounced wear processes in engine components.
5. The data suggest a nonlinear pattern in the depletion of Phos. antiwear additives, with the rate of depletion appearing to accelerate as the recommended oil change interval is further exceeded. For example, after exceeding the interval by 20,000 km, the depletion level was 73% higher than after a 5,000 km exceedance.
6. Statistical analysis indicated notable differences between the tested oils, particularly for sample OP_2384, which exhibited poorer tribological properties despite not exceeding the recommended change interval. These findings are based on a limited dataset and should be interpreted accordingly.

The case of bus OP_2384 underscores the value of systematically analyzing service data and identifying patterns that could be associated with potential failures, supporting a more proactive approach to fleet maintenance. The combined use of tribological testing (four-ball apparatus), FTIR spectroscopy, and XRF analysis was effective in associating various indicators with engine oil degradation and identifying key wear markers in the samples studied. The results indicate that oil degradation becomes more pronounced after exceeding the recommended interval by 10,000–15,000 km. Exceeding this by more than 15,000–20,000 km was associated with a marked increase in engine component wear, as evidenced by the increased iron content (131.67 ppm) in the analyzed oil, which may be linked to a higher risk of serious failures. This study presents a holistic approach that delivers specific, quantifiable thresholds for extended oil change intervals, facilitating practical implementation in vehicle fleet management.

## Limitations of the study

The study was conducted on a limited number of samples (5 buses), which affects the reliability of statistical analyses and the generalizability of the results. All tested buses were of the same model (Autosan Sancity M12LF), limiting the ability to generalize findings to other vehicle and engine types. The study did not account for detailed operational condition data, such as engine load, idle time, or frequency of stops, which could influence the results. However, since the analyzed vehicles operated within the same urban area, albeit on different transit routes, it can be assumed that their operating conditions were similar. The lack of engine operating temperature monitoring during operation prevented an assessment of the impact of thermal loads on oil degradation. Additionally, the research methodology did not include an analysis of the size and morphology of abrasive metal particles, which could provide further insights into wear mechanisms.

Given these limitations, future research directions will involve expanding the study to a larger and more diverse vehicle fleet, including various bus models and engine types, to enhance the representativeness of the results. Furthermore, comparative analyses of different engine oil types (mineral, semi-synthetic, and synthetic) under identical operating conditions are planned to evaluate the impact of oil base type on the durability of performance-enhancing additives. In the longer term, continuous monitoring of oil parameters during bus operation using OBD recorders is planned, enabling correlation of oil degradation with real-world operating conditions. Based on this, a mathematical model will be developed to predict the rate of oil degradation based on operating conditions, facilitating the optimization of oil change intervals for individual vehicles.

## Data Availability

The data presented in this study are available on request from the corresponding author.
